# Supplementary material for: Influence maximization in Boolean networks
Source: Nat Commun. 2022 Jun 16;13:3457. doi: 10.1038/s41467-022-31066-0 (PMC9203747; doi:10.1038/s41467-022-31066-0)
Supplement: Supplementary file 1 — Supplementary Information [file 41467_2022_31066_MOESM1_ESM.pdf]

Supplementary Information "Influence maximization in Boolean networks"

Thomas Parmer,[1] Luis M. Rocha,[2,3] and Filippo Radicchi[1,*]

[1] *Center for Complex Networks and Systems Research,*
Luddy School of Informatics, Computing, and Engineering,
Indiana University, Bloomington, Indiana 47408, USA
[2] *Consortium for Social and Biomedical Complexity,*
Systems Science and Industrial Engineering Department,
Thomas J. Watson College of Engineering and Applied Science,
Binghamton University (State University of New York), Binghamton, New York 13902, USA
[3] *Instituto Gulbenkian de Ciência, Oeiras 2780-156, Portugal*

* Corresponding author: filiradi@indiana.edu

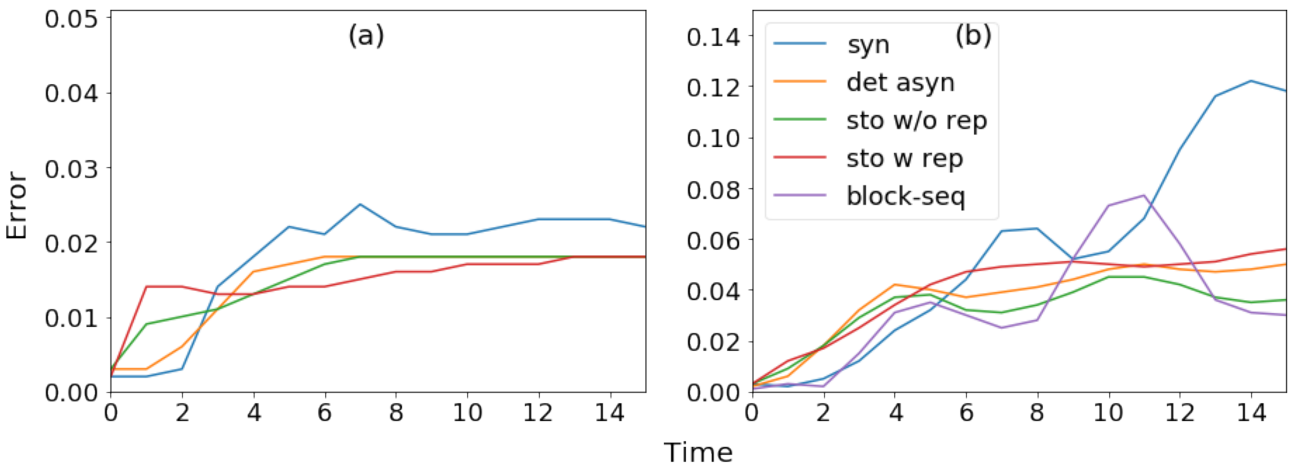

FIG. S1. **Accuracy of the individual-based mean-field approximation across various update schedules.** (a) We evaluate the error $e(t)$, as defined in Eq. (5) of the main text, committed by the individual-based mean-field approximation (IBMFA) in predicting the ground-truth configuration of the system at stage t of the dynamics. Each curve corresponds to a different updating schedule in the *Drosophila melanogaster* segment polarity network (SPN): synchronous (syn), deterministic asynchronous (det asyn), stochastic asynchronous without replacement (sto w/o rep) and stochastic with replacement (sto w rep). In all asynchronous updating schemes, the state of a single node is updated while the state of all other nodes is kept invariant, and one unit of time corresponds to a number of updates equal to the system size. The node whose state is updated is selected in different ways depending on the updating scheme: in (det asyn), the node is selected according to a predetermined sequence; in (sto w rep), the node is selected at random among all the possible nodes; in (sto w/o rep), the node is selected according to a sequence, but such a sequence is randomized at every time step. All results are averaged over $R = 100$ independent simulations of $M = 100$ randomly selected updating schemes. Initial configurations are sampled from Eq. (3) of the main paper, where the probability of node i to be active equals $s_i(t = 0) = 1/2$ for all $i = 1, \ldots, N$. (b) Same as in panel a, but for the yeast cell cycle network. In addition to the other update schedules, the block-sequential update (block-seq) from Ref. [2] is included. In this case, nodes are updated sequentially in blocks, where all nodes in the same block are updated synchronously. The blocks updated in order are: ['CellSize','Swi5','Cdc20/14','Clb5,6'] , ['MBF','Sic1'] , ['Cln3','SBF','Clb1,2','Mcm1/SFF'], ['Cln1,2'], and ['Cdh1']. One unit of time is given by a full update of all blocks.

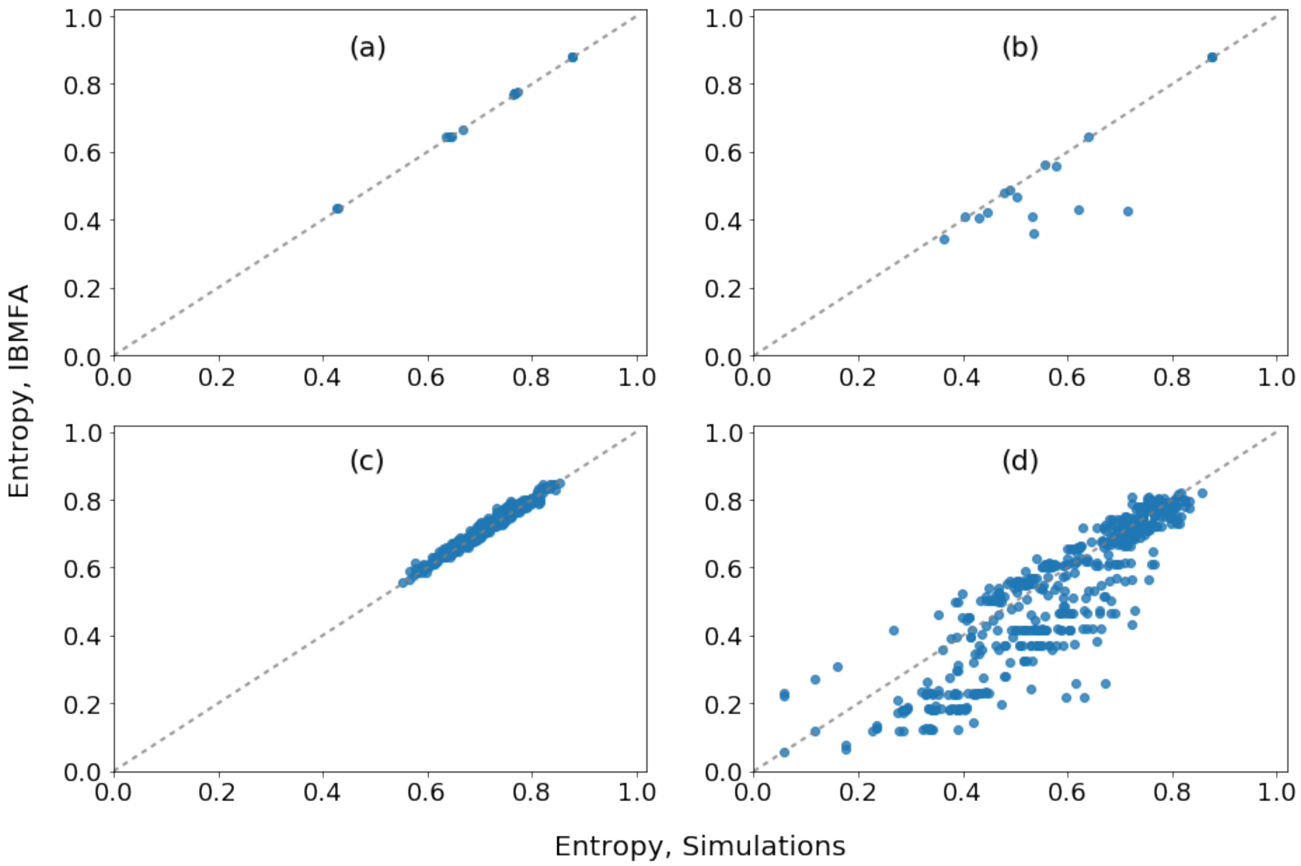

FIG. S2. **Average node and configuration entropies for the *Drosophila melanogaster* segment polarity network.** (a) We consider all possible seed sets of size $|\mathcal{X}| = 2$. We then compare the residual entropy $h_2(s)$ of each node in the network after $t = 1$ iterations of the IBMFA, averaged across all seed sets, to the residual entropy of each node averaged over $R = 100$ simulations of each seed set. Entropy is measured in bits; maximum entropy per node is 1 bit. Different dots correspond to different nodes in the network. The dashed line indicates perfect agreement between entropy estimates. (b) Same as in panel a, but for iteration $t = 10$. (c) We again consider all possible seed sets of size $|\mathcal{X}| = 2$. We then compare the residual entropy $H(\vec{s}|\mathcal{X})$ of the network configuration after $t = 1$ iterations of the IBMFA, averaged across all seed sets, to the residual entropy of the network configuration averaged over $R = 100$ simulations of each seed set. Entropy is measured in bits and is normalized by the size of the network. Different dots correspond to different choices for the set of seed nodes $\mathcal{X}$. (d) Same as in panel c, but for iteration $t = 10$. The results show a strong positive correlation between our method and the simulations, although after 10 iterations we see that the IBMFA tends to underestimate entropy as compared to the simulations.

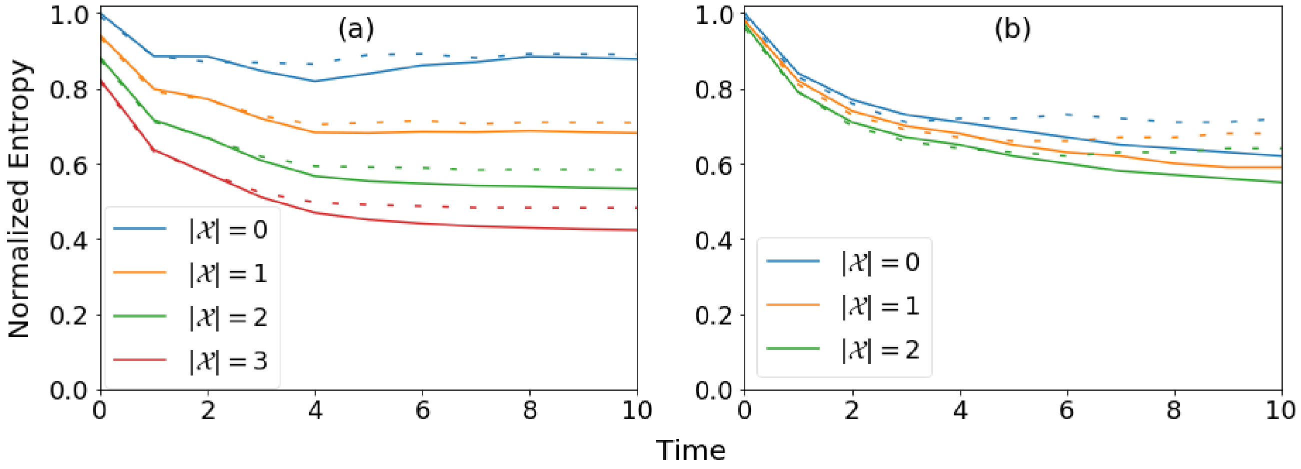

FIG. S3. **Entropies of the IBMFA compared to simulations in genetic regulatory networks.** (a) We monitor the residual entropy of the *Drosophila melanogaster* segment polarity network as a function of time. Entropy is measured in bits and is normalized by the size of the network, and then averaged over all possible seed sets of size $|\mathcal{X}|$. Full curves correspond to predictions made by the IBMFA, while dashed curves correspond to the average entropy based on $R = 100$ independent simulations of each possible seed set. (b) Same as in panel a, but for the T-LGL leukemia network. Although node entropies $h_2(s)$ are sometimes lower and sometimes higher than the simulations (and can increase as well as decrease over time), the IBMFA on average consistently predicts lower entropy of the network configuration $H(\vec{s}|\mathcal{X})$ than the simulations.

Attractor	Method	Driver set
1	BF	{CellSize : 0; SBF : 0; MBF : 0; Sic1 : 1; Cdh1 : 1}
	GR	{CellSize : 0; SBF : 0; MBF : 0; Sic1 : 1; Cdh1 : 1}
2	BF	{CellSize : 1; SBF : 1; MBF : 1; Sic1 : 0; Clb5,6 : 0; Clb1,2 : 1}
	GR	{CellSize : 1; SBF : 1; MBF : 1; Sic1 : 0; Clb5,6 : 0; Clb1,2 : 1}
3	BF	{CellSize : 1; SBF : 1; MBF : 1; Clb5,6 : 1; Clb1,2 : 1}
	GR	{CellSize : 1; SBF : 1; MBF : 1; Clb5,6 : 1}
4	BF	{CellSize : 0; SBF : 0; Sic1 : 0; Cdh1 : 0; Mcm1/SFF : 0}
	GR	{CellSize : 0; SBF : 0; MBF : 0; Sic1 : 0; Clb5,6 : 0; Cdh1 : 0; Clb1,2 : 0}
5	BF	{CellSize : 1; SBF : 1; MBF : 0; Sic1 : 0; Clb1,2 : 1}
	GR	{CellSize : 1; SBF : 1; MBF : 0; Sic1 : 0; Clb1,2 : 1}
6	BF	{CellSize : 0; SBF : 1; Mcm1/SFF : 0}
	GR	{CellSize : 0; SBF : 1; MBF : 0; Clb5,6 : 0; Clb1,2 : 0}
7	BF	{CellSize : 0; SBF : 0; MBF : 1; Sic1 : 1; Cdh1 : 1}
	GR	{CellSize : 0; SBF : 0; MBF : 1; Sic1 : 1; Clb5,6 : 0; Cdh1 : 1}
8	BF	{CellSize : 1; SBF : 0; MBF : 1; Sic1 : 0; Clb5,6 : 1; Clb1,2 : 1}
	GR	{CellSize : 1; SBF : 0; MBF : 1; Sic1 : 0; Clb5,6 : 1}
9	BF	{CellSize : 0; SBF : 0; MBF : 1; Sic1 : 1; Cdh1 : 0}
	GR	{CellSize : 0; SBF : 0; MBF : 1; Sic1 : 1; Clb5,6 : 0; Cdh1 : 0}
10	BF	{CellSize : 0; SBF : 0; MBF : 0; Sic1 : 0; Cdh1 : 1}
	GR	{CellSize : 0; SBF : 0; MBF : 0; Sic1 : 0; Clb5,6 : 0; Cdh1 : 1}
11	BF	{CellSize : 0; SBF : 0; MBF : 0; Sic1 : 1; Cdh1 : 0}
	GR	{CellSize : 0; SBF : 0; MBF : 0; Sic1 : 1; Cdh1 : 0}

TABLE S1. **Accuracy of the greedy selection process to predict ground-truth minimal driver sets in the yeast cell-cycle network.** Driver sets calculated by brute-force computation (BF) compared to our greedy selection algorithm (GR) for the yeast cell-cycle network. The attractors are in the same order as in Fig. S9. For each attractor, we specify the label of the driver nodes and their states. For sake of compactness, we use a different notation with respect to the rest of the paper. For example, the notation $\{\mathrm{CellSize} : 0; \mathrm{SBF} : 0; \mathrm{MBF} : 0; \mathrm{Sic1} : 1; \mathrm{Cdh1} : 1\}$ corresponds to the set $\{(\mathrm{CellSize}, \hat{\sigma}_{\mathrm{CellSize}} = 0), (\mathrm{SBF}, \hat{\sigma}_{\mathrm{SBF}} = 0), (\mathrm{MBF}, \hat{\sigma}_{\mathrm{MBF}} = 0), (\mathrm{Sic1}, \hat{\sigma}_{\mathrm{Sic1}} = 1), (\mathrm{Cdh1}, \hat{\sigma}_{\mathrm{Cdh1}} = 1)\}$. Attractor 1 corresponds to the G1 phase of the cell cycle and is the attractor with the largest basin.

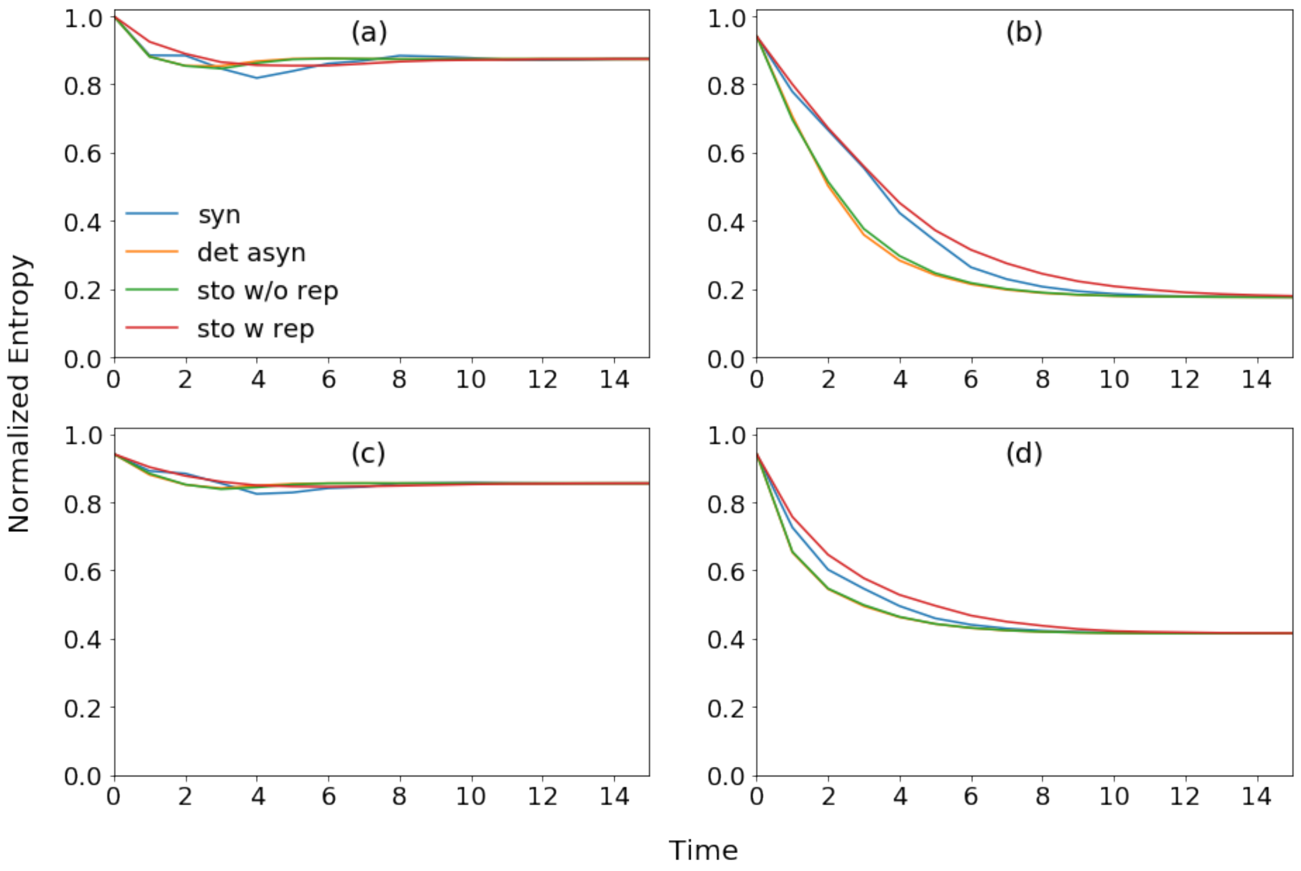

FIG. S4. **Dynamical influence in the *Drosophila Melanogaster* SPN under different update schedules.** (a) We monitor the residual entropy of the *Drosophila melanogaster* segment polarity network (SPN) as a function of time. Entropy is measured in bits and is normalized by the size of the network. We report results for an empty seed set, i.e., for $\mathcal{X} = \emptyset$. Different curves correspond to different choices of the update schedule: synchronous (syn), deterministic asynchronous (det asyn), stochastic asynchronous without replacement (sto w/o rep) and stochastic with replacement (sto w rep). For definitions of the updating schemes, see caption of Figure S1. All results are averaged over $R = 100$ independent simulations of $M = 100$ models. (b) Same as in panel a, but for $\mathcal{X} = \{(\text{en}, \hat{\sigma}_{\text{en}} = 1)\}$. (c) Same as in panel a, but for $\mathcal{X} = \{(\text{CIR}, \hat{\sigma}_{\text{CIR}} = 0)\}$. (d) Same as in panel a, but for $\mathcal{X} = \{(\text{CIR}, \hat{\sigma}_{\text{CIR}} = 1)\}$.

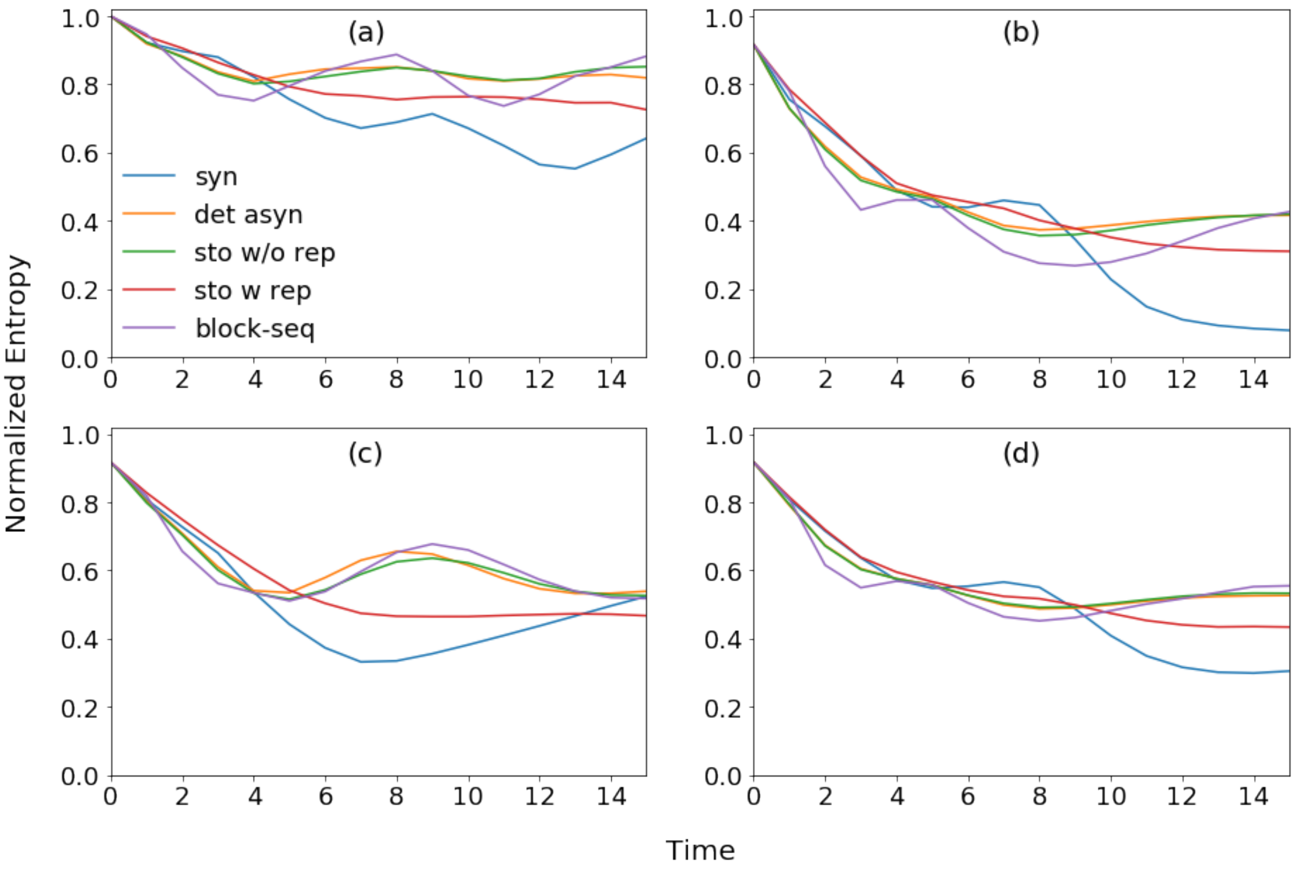

FIG. S5. **Dynamical influence in the yeast cell-cycle network under different update schedules.** (a) We monitor the residual entropy of the yeast cell-cycle network as a function of time. Entropy is measured in bits and is normalized by the size of the network. We report results for $\mathcal{X} = \emptyset$. Different curves correspond to different choices for the update schedule: synchronous (syn), deterministic asynchronous (det asyn), stochastic asynchronous without replacement (sto w/o rep), stochastic with replacement (sto w rep), and block-sequential (block-seq). For definitions of the updating schemes, see caption of Figure S1. All results are averaged over $R = 100$ independent simulations of $M = 100$ models. (b) Same as in panel a, but for $\mathcal{X} = \{(\text{CellSize}, \hat{\sigma}_{\text{CellSize}} = 1)\}$. (c) Same as in panel a, but for $\mathcal{X} = \{(\text{Cln3}, \hat{\sigma}_{\text{Cln3}} = 0)\}$. (d) Same as in panel a, but for $\mathcal{X} = \{(\text{Cln3}, \hat{\sigma}_{\text{Cln3}} = 1)\}$.

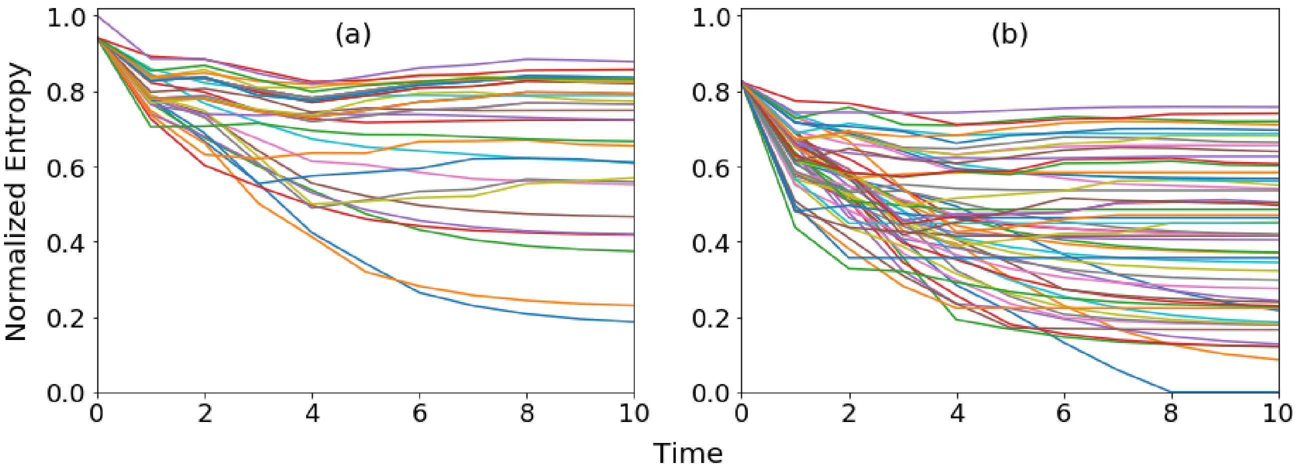

FIG. S6. **Dynamical influence in the *Drosophila melanogaster* segment polarity network.** (a) We monitor the residual entropy of the network as a function of time. Entropy is measured in bits and is normalized by the size of the network. Different curves correspond to different choices for the set of seed nodes $\mathcal{X}$. All possible seed sets of size $|\mathcal{X}| = 1$ are shown. (b) Same as in panel a, but a random sample of 1/100 of all possible seed sets of size $|\mathcal{X}| = 3$ are shown; one of the seed sets results in zero entropy (an attractor of the system).

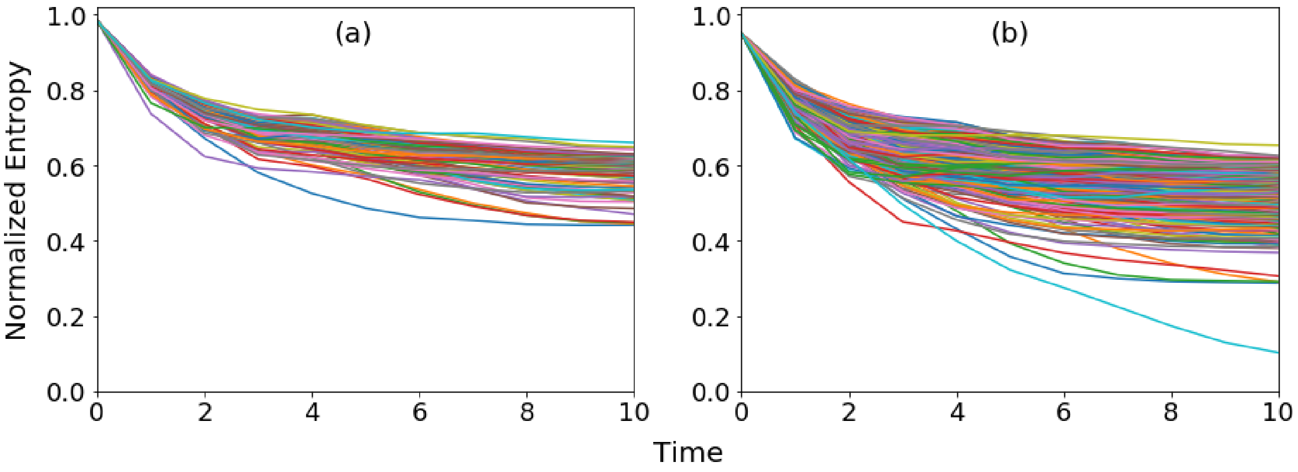

FIG. S7. **Dynamical influence in the T-LGL leukemia network.** (a) We monitor the residual entropy of the network as a function of time. Entropy is measured in bits and is normalized by the size of the network. Different curves correspond to different choices for the set of seed nodes $\mathcal{X}$. All possible seed sets of size $|\mathcal{X}| = 1$ are shown. (b) Same as in panel a, but a random sample of 1/1000 of all possible seed sets of size $|\mathcal{X}| = 3$ are shown.

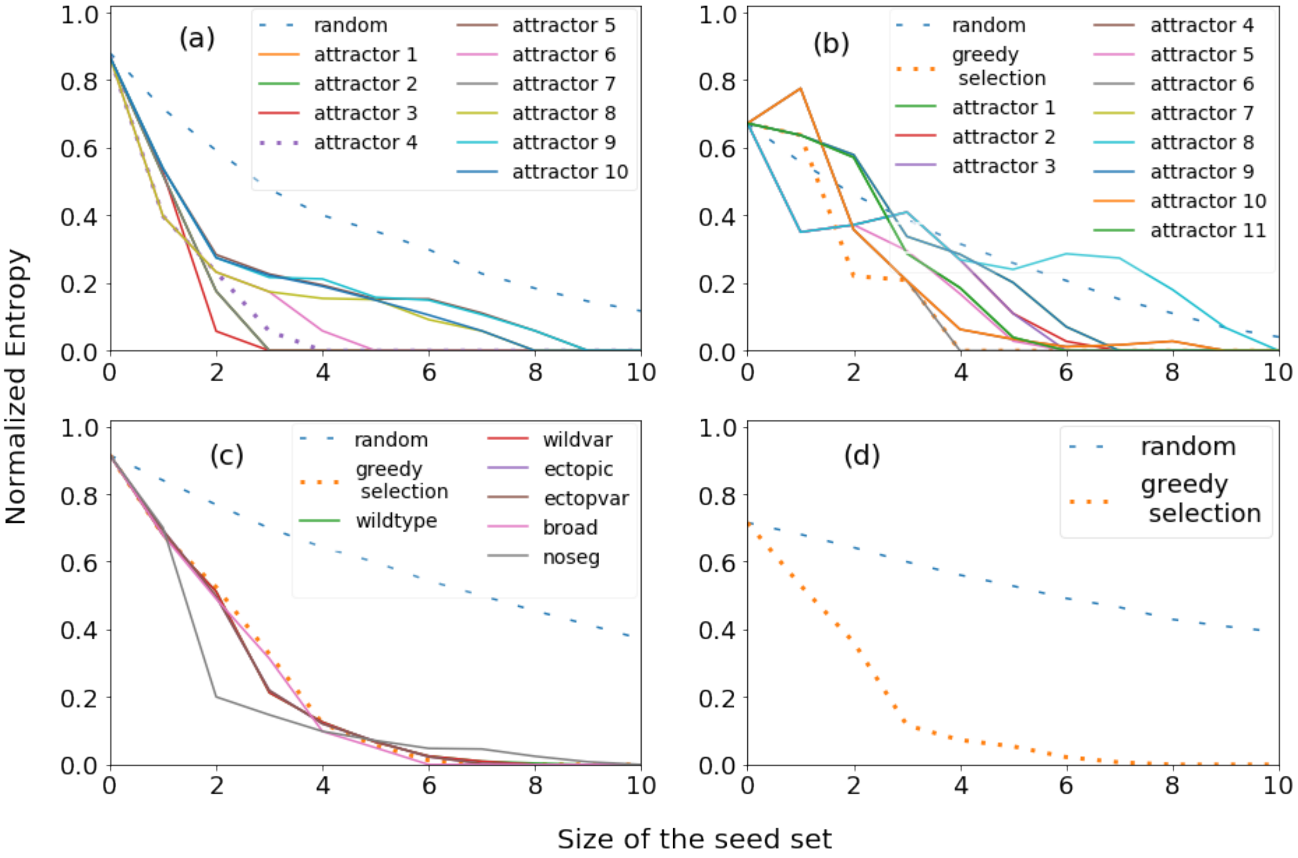

FIG. S8. **Driving genetic regulatory networks to a desired attractor.** (a) Residual entropy as a function of the size of the seed set for seed sets leading to the 10 attractors in the *Drosophila melanogaster* segment polarity network (the size of the network is $N = 17$). The values shown are based on averaging $R = 100$ simulations of the selected seeds. Circles denote the greedy selection process towards an unconstrained attractor, which in this case finds attractor 4. As a term of comparison, we display also the curve corresponding to seed sets composed of randomly selected nodes' indices/states (dashed line). The curve displays the value of the entropy averaged over $R = 100$ simulations of 100 random seed sets. The set size indicated does not take into account seed reduction which results in driver sets of size $|\mathcal{X}| = 3$ or $|\mathcal{X}| = 4$ for each attractor in the network. (b) Same as panel a, but for the yeast cell-cycle network ($N = 12$). Solid lines show greedy selection towards each of the 11 attractors in the network. The unconstrained greedy selection process in this case finds an attractor that does not exist if nodes are not pinned. Interestingly, entropy must increase during the seed selection process to reach some of the attractors. (c) Same as in panel a, but for the *Drosophila melanogaster* parasegment network ($N = 60$). Solid lines show greedy selection towards six biologically relevant attractors, representing the wildtype phenotype, wildtype variant (wildvar), ectopic phenotype, ectopic variant (ectopvar), broad stripes (broad), and no segmentation (noseg). Even though this network is too large for the attractor landscape to be fully described, we are able to find each of these known attractors by pinning as few as six to eleven nodes. (d) Same as in panel a, but for the T-LGL leukemia network ($N = 60$). Entropy decreases quickly after the selection of only three nodes and falls to zero after nine selections.

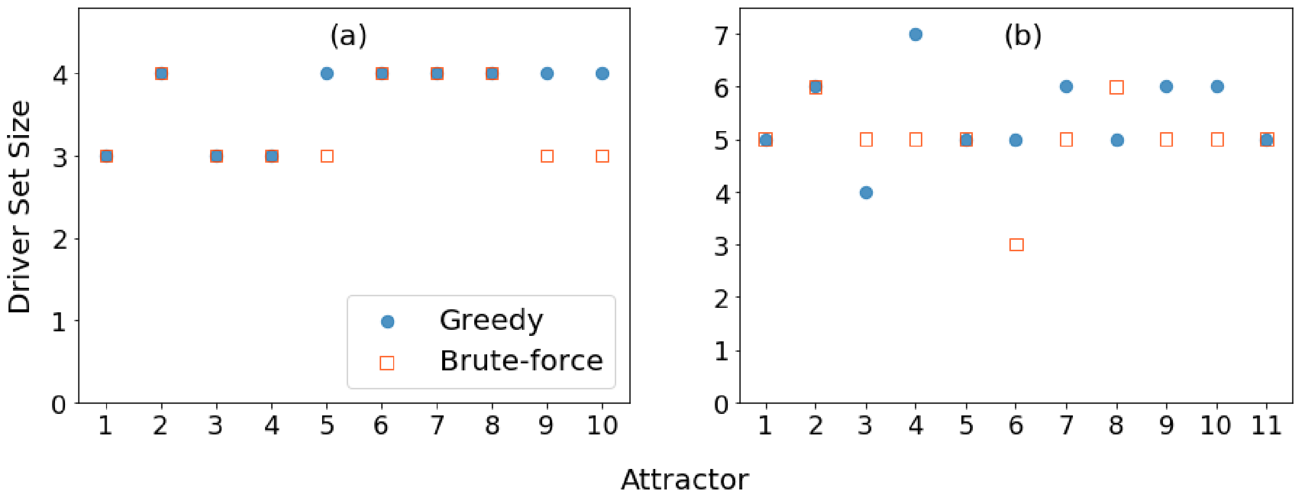

FIG. S9. **Accuracy of the greedy selection process to predict ground-truth minimal driver sets**. (a) We use a brute-force approach on the state transition graph to determine the ground-truth size of the minimal driver set for each of the 10 attractors of the *Drosophila melanogaster* segment polarity network (SPN) (orange squares). Minimal driver sets to the same attractors are approximated using our constrained greedy optimization method (blue circles). (b) Same analysis as in panel (a), but for the 11 fixed points of the yeast *Saccharomyces cerevisiae* cell-cycle network.

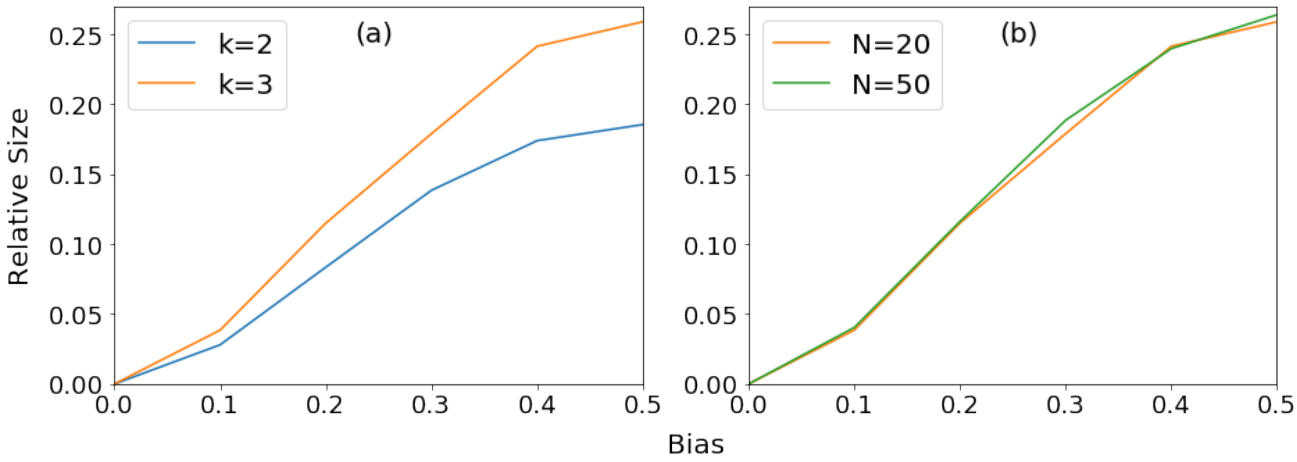

FIG. S10. **Driver set sizes in random Boolean network models with different biases.** (a) We consider random Boolean networks (RBNs) where the output of the lookup table is chosen equal to 0 with probability equal $0 \leq B \leq 1/2$. We identify the optimal sets of drivers by implementing our greedy strategy on RBNs with network size $N = 20$ and fixed degree k. We plot the relative size of the optimal driver set as a function of the bias B. Different curves correspond to different k values. Results refer to average values over 100 RBNs with the same bias. (b) Same as panel a, except with variable network size N and fixed degree $k = 3$. Different curves correspond to different N values; note that the $k = 3$ line in panel a is the same as the $N = 20$ line in panel b.

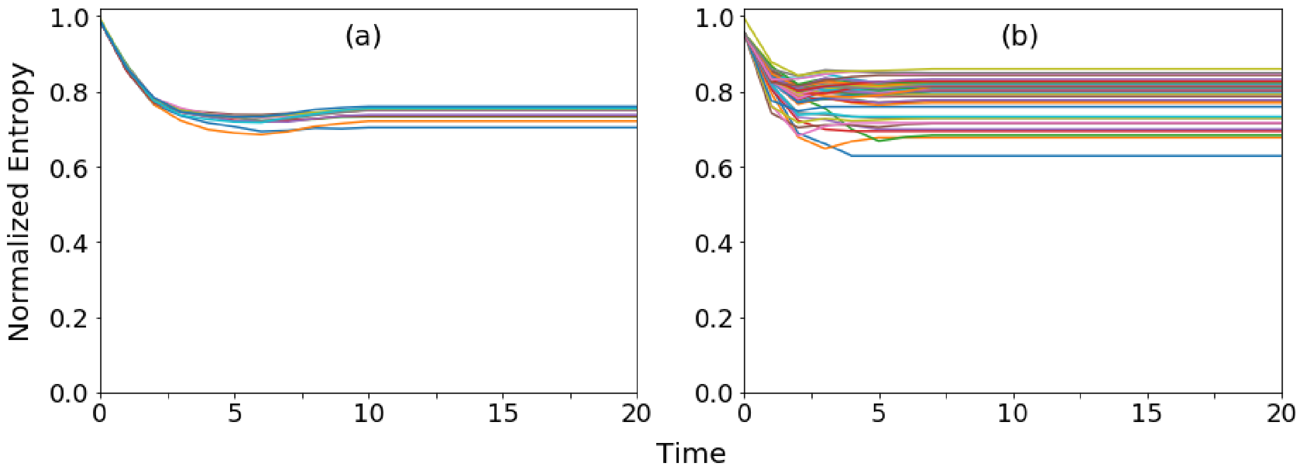

FIG. S11. **Dynamical influence in the CD4 T Cell Signaling network and the HH Drosophila Signaling Pathway.** (a) We monitor the residual entropy of the CD4 T Cell Signaling network [1] (N=188, the largest network shown in Fig. 4) as a function of time. Entropy is measured in bits and is normalized by the size of the network. Different curves correspond to different choices for the set of seed nodes $\mathcal{X}$. A random sample of 10 of the possible seed sets of size $|\mathcal{X}| = 1$ are shown. (b) Same as in panel a, but for the HH Drosophila Signaling Pathway [3] (N=24, the network with the largest relative driver set size in Fig. 4). All possible seed sets of size $|\mathcal{X}| = 1$ are shown. For both networks, entropy values at $T = 10$ iterations of the IBMFA represent well the long-term entropy values of the system.

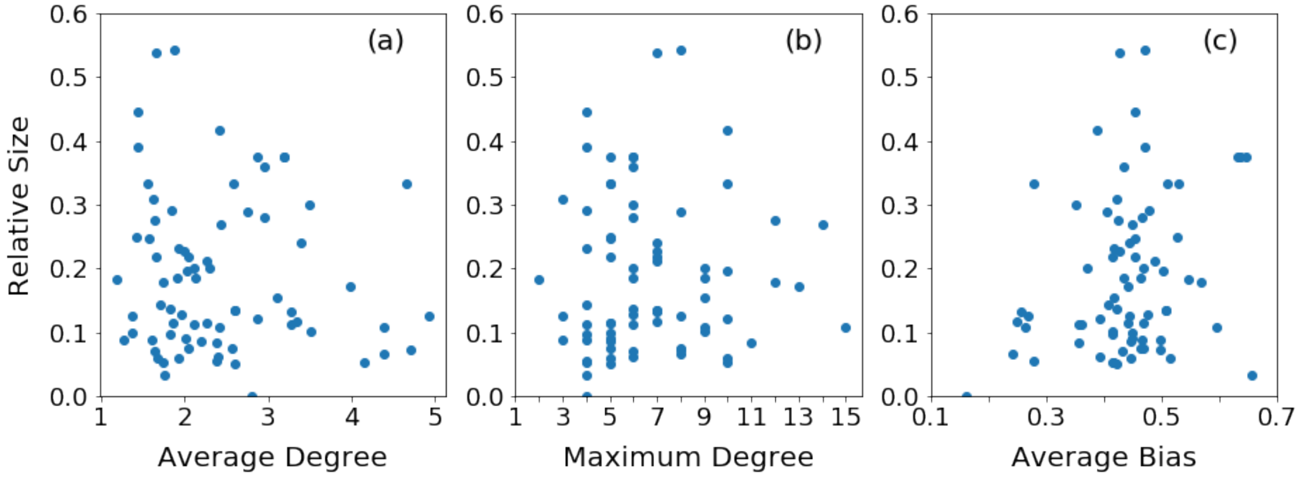

FIG. S12. **Driver set statistics in the Cell Collective.** We identify the optimal sets of drivers by implementing our greedy strategy on the 74 networks that are part of the Cell Collective repository. The relative size of the identified driver set is plotted against the average degree (a), the maximum degree (b), and the average bias of the lookup tables (c) of the corresponding network. There is one driver set per network. The results show that driver set size does not correlate well with the network's average degree (Pearson's R=-0.10, p=0.41), maximum degree (Pearson's R=0.03, 0.78), or average bias (Pearson's R=0.26, 0.03).

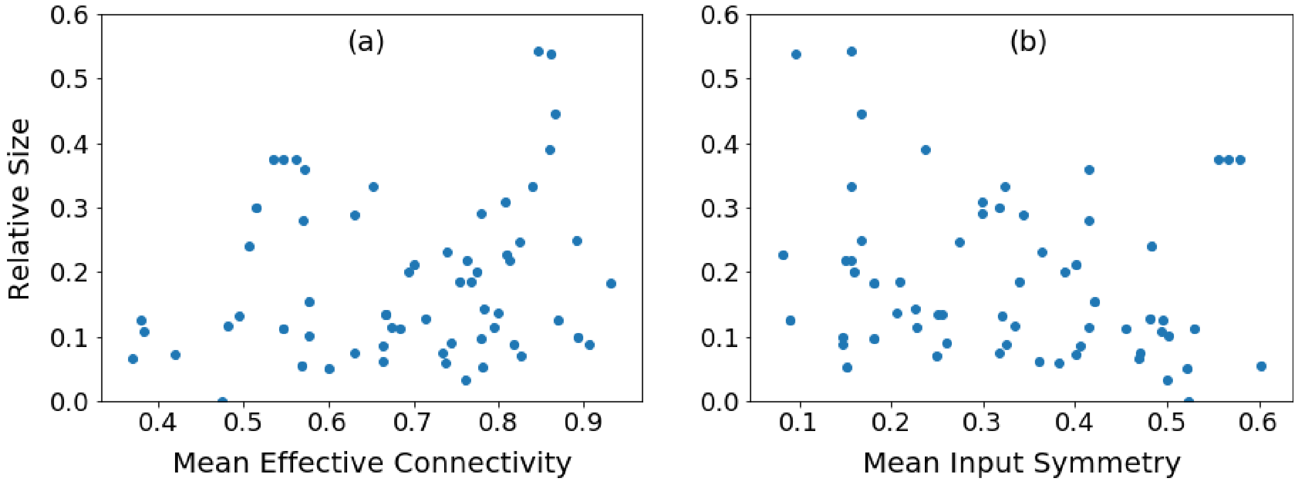

FIG. S13. **Driver set canalization statistics in the Cell Collective.** We identify the optimal sets of drivers by implementing our greedy strategy on the 62 networks that are part of the Cell Collective repository and have a maximum degree $k_{max} < 10$. The relative size of the identified driver set is plotted against the average effective connectivity (a) and the average input symmetry (b) of the corresponding network, using upper bounds to calculate an individual node's input redundancy (the complement of its effective connectivity) and input symmetry [4]. There is one driver set per network. The results show that driver set size does not correlate well with the network's average effective connectivity (Pearson's R=0.20, p=0.11) or average input symmetry (Pearson's R=-0.22, 0.09).

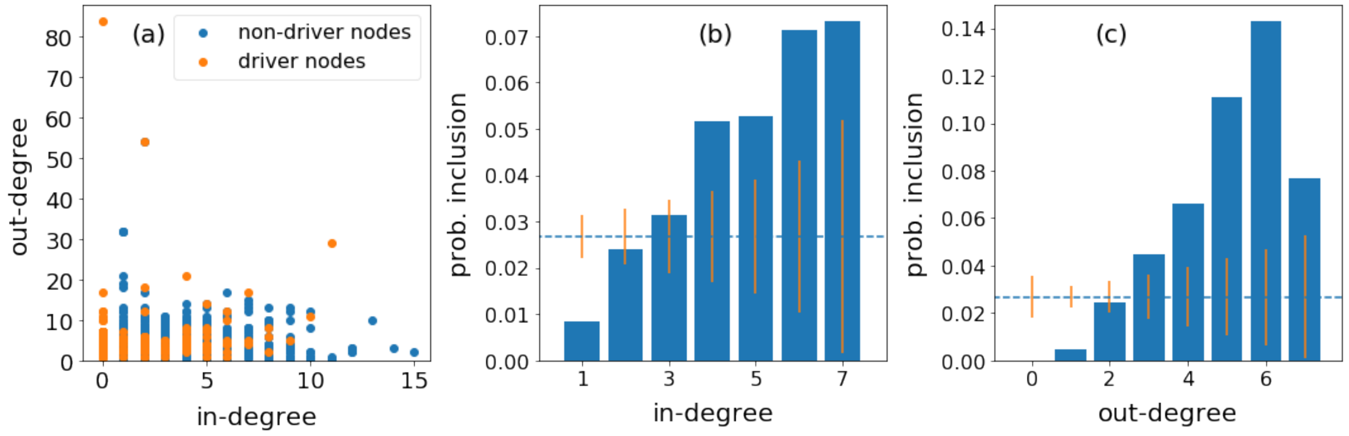

FIG. S14. **Topological centrality of nodes in the minimal driver sets.** Results are for nodes in all 74 networks in the Cell Collective repository. (a) Each node is represented by its in- and out-degree. We display all nodes from all networks of the repository. We color points depending on whether the node is identified within the minimal driver set by the unconstrained greedy selection algorithm. (b) Conditional probability of a node to be part of the minimal driver set depending on its in-degree. We excluded from the analysis all nodes with null in-degree as they are always identified as drivers. The dashed blue line indicates the expected probability with no dependence on the in-degree; error bars indicate one standard deviation away from the expected value in such a null model. (c) Conditional probability of a node to be part of the minimal driver set depending on its out-degree. Reference value and error bars are obtained in the same way as for panel b.

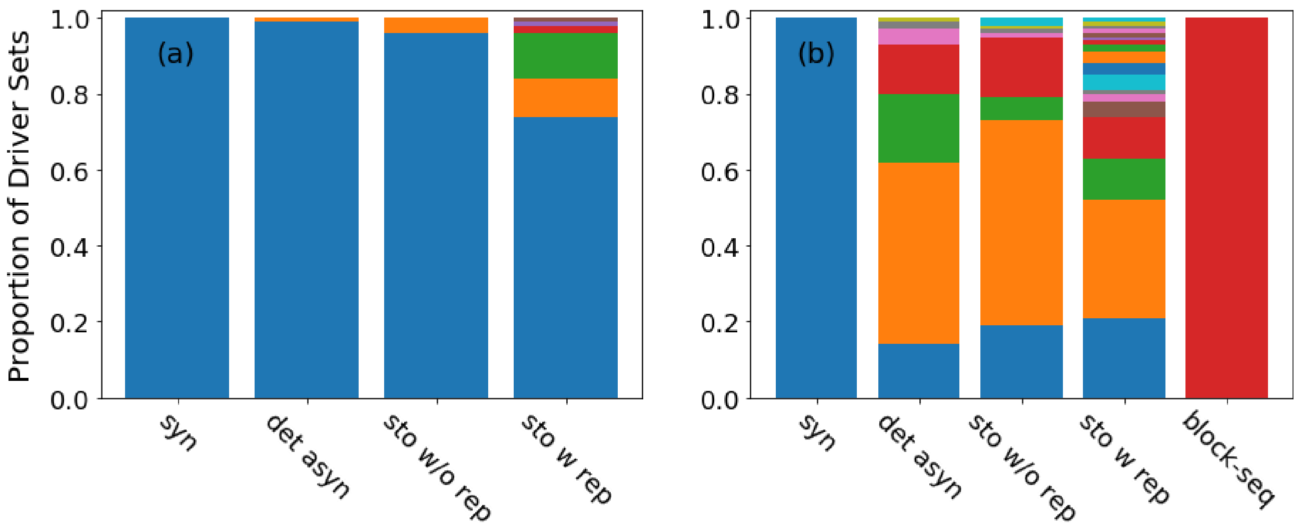

FIG. S15. **Attractor found by the greedy algorithm under different updating schemes.** (a) We apply our unconstrained optimization algorithm for finding the minimal driver set toward an unspecified fixed-point of the *Drosophila melanogaster* segment polarity network (SPN). We consider different choices for the update schedule: synchronous (syn), deterministic asynchronous (det asyn), stochastic asynchronous without replacement (sto w/o rep) and stochastic with replacement (sto w rep). We measure the frequency of observation of attractor 4 (blue) for $M = 100$ models of the various updating schemes. Different attractors, each identified by a different color, are reached only for a few realizations of the asynchronous updating rules. (b) Same analysis as in panel a, but for the yeast *Saccharomyces cerevisiae* cell-cycle network. In this case, several different attractors, each identified by a different color, are reached depending on the updating scheme. We included in the analysis also the block-sequential (block-seq) updating scheme.

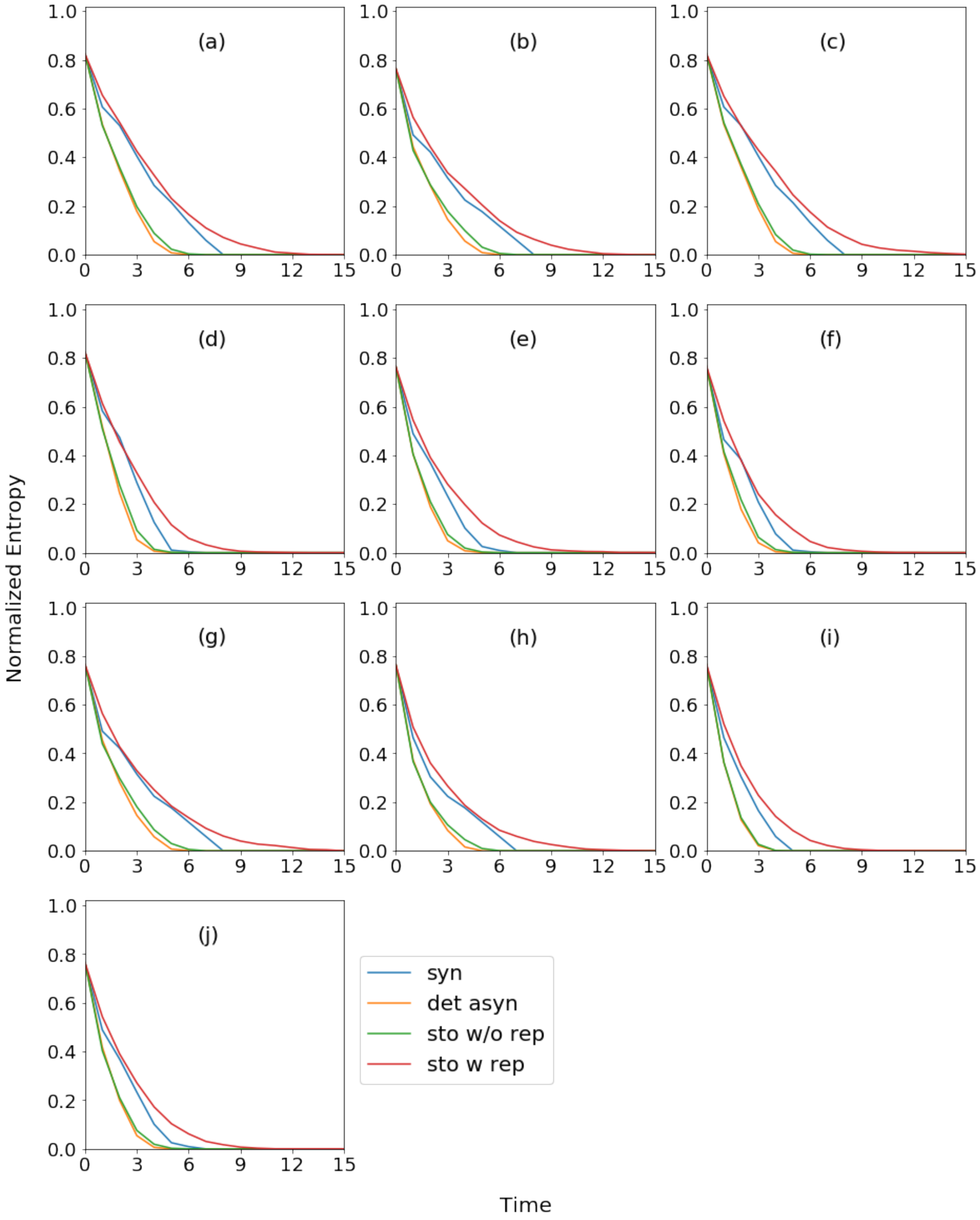

FIG. S16. **Driving the *Drosophila Melanogaster* SPN to a desired attractor under different update schedules.** (a) We monitor the residual entropy of the *Drosophila Melanogaster* segment polarity network (SPN) as a function of time. Entropy is measured in bits and is normalized by the size of the network. We report results for the driver set solution for attractor 1 found by our greedy approximation algorithm under synchronous updating. Different curves correspond to different choices for the update schedule: synchronous (syn), deterministic asynchronous (det asyn), stochastic asynchronous without replacement (sto w/o rep) and stochastic with replacement (sto w rep). All results are averaged over $R = 100$ independent simulations of $M = 100$ models. (b-j) Same as in panel a, but for attractors 2-10. The attractors are displayed in the same order as Fig. 3 in the main text.

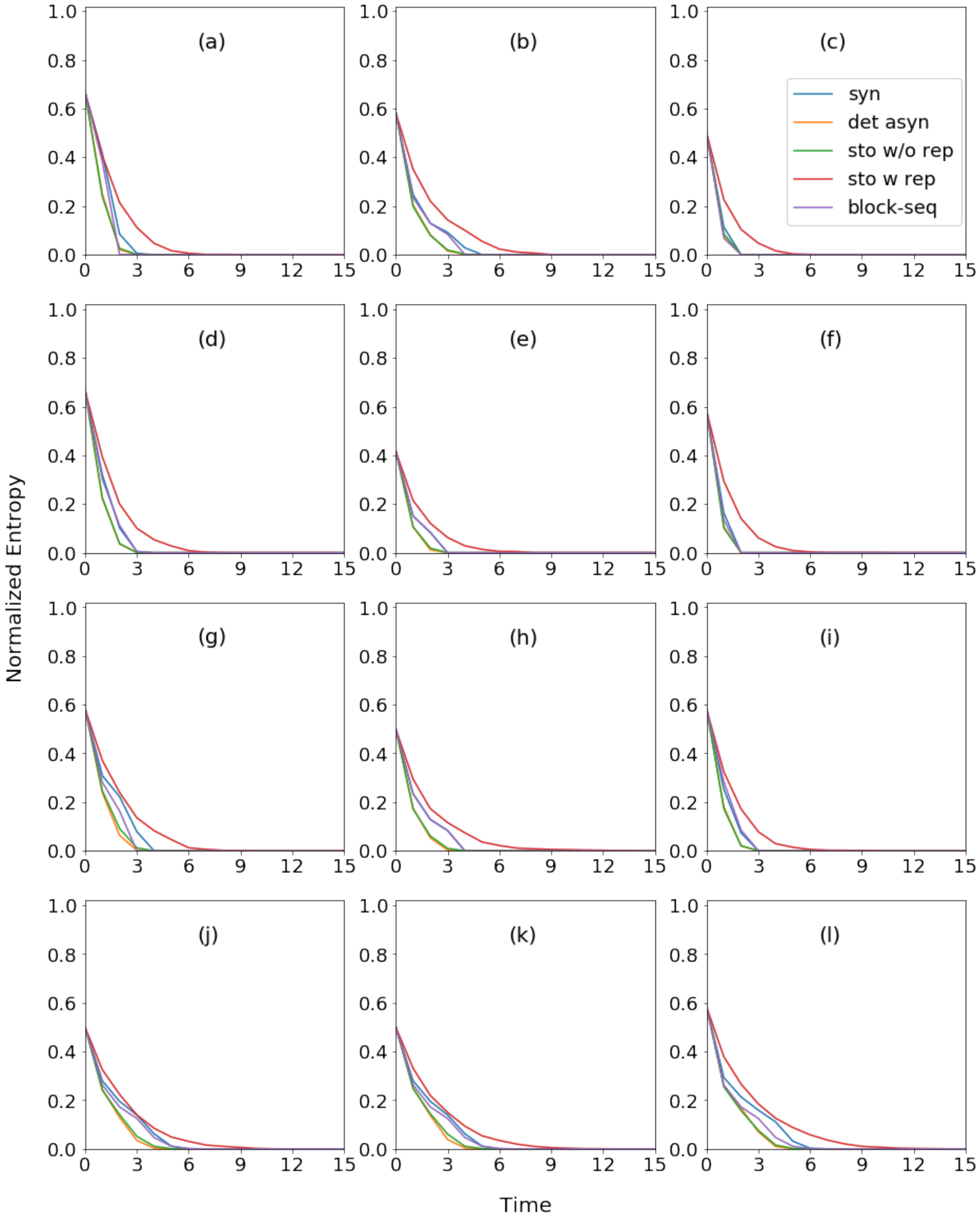

FIG. S17. **Driving the yeast *Saccharomyces cerevisiae* cell-cycle network to a desired attractor under different updating schedules.** (a) We monitor the residual entropy of the yeast cell-cycle network as a function of time. Entropy is measured in bits and is normalized by the size of the network. We report results for the driver set solution for the attractor found by our unconstrained greedy approximation algorithm under synchronous updating. Different curves correspond to different choices for the update schedule: synchronous (syn), deterministic asynchronous (det asyn), stochastic asynchronous without replacement (sto w/o rep), stochastic with replacement (sto w rep), and block-sequential (block-seq). (b-l) Same as in panel a, but for the original 11 attractors of the yeast cell-cycle network. The attractors are displayed in the same order as in Fig. S8.

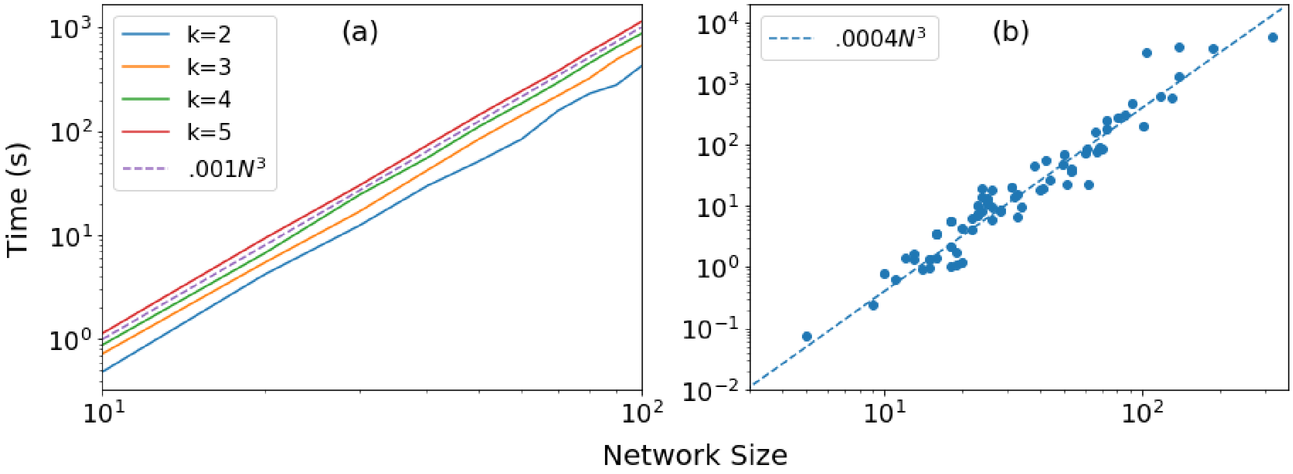

FIG. S18. **Time to find driver sets in Boolean networks.** (a) We identify the optimal sets of drivers by implementing our greedy strategy on random Boolean networks (RBNs) with variable network size N and fixed degree k. We plot the average time of computation to find one driver set as a function of the network size. Different curves correspond to different k values. Results refer to average values over 10 RBNs. The time to find driver sets increases with k. (b) Optimal driver sets are identified by our algorithm for the 74 networks in the Cell Collective repository. The time to find each network's driver set is plotted against the network size. Each dot represents one network. The time to find driver sets is exponential in k, but most cell collective networks are sparse (average degree ≈ 2). In both plots, reference cubic functions are shown as dashed curves. Computations were performed using an Intel Core i7 3.2 GHz processor.

SUPPLEMENTARY REFERENCES

[1] Conroy, B.D. *et al.*, Design, Assessment, and in vivo Evaluation of a Computational Model Illustrating the Role of CAV1 in CD4(+) T-lymphocytes. *Front. Immunol.* **5**, 599 (2014).

[2] Goles, E., Montalva, M. & Ruz, G. A. Deconstruction and dynamical robustness of regulatory networks: application to the yeast cell cycle networks. *B. Math. Biol.* **75**, 939–966 (2013).

[3] Abibatou Mbodj, A., Junion, G., Brun, C., Furlong, E. E. M. & Thieffry, D. Logical modelling of Drosophila signalling pathways. *Mol. Biosyst.* **9**, 2248—2258 (2013).

[4] Marques-Pita, M. & Rocha, L. M. Canalization and control in automata networks: body segmentation in drosophila melanogaster. *PloS One* **8**, e55946 (2013).
