# Supplementary material for: Influence maximization in Boolean networks
Source: Nat Commun. 2022 Jun 16;13:3457. doi: 10.1038/s41467-022-31066-0 (PMC9203747; doi:10.1038/s41467-022-31066-0)
Supplement: Supplementary file 3 — Reporting Summary [file 41467_2022_31066_MOESM3_ESM.pdf]

nature portfolio

Corresponding author(s): Filippo Radicchi

Last updated by author(s): May 22, 2022

Reporting Summary

Nature Portfolio wishes to improve the reproducibility of the work that we publish. This form provides structure for consistency and transparency in reporting. For further information on Nature Portfolio policies, see our Editorial Policies and the Editorial Policy Checklist.

Statistics

For all statistical analyses, confirm that the following items are present in the figure legend, table legend, main text, or Methods section.

n/a	Confirmed	
☐	☒	The exact sample size (*n*) for each experimental group/condition, given as a discrete number and unit of measurement
☒	☐	A statement on whether measurements were taken from distinct samples or whether the same sample was measured repeatedly
☐	☒	The statistical test(s) used AND whether they are one- or two-sided<br>*Only common tests should be described solely by name; describe more complex techniques in the Methods section.*
☒	☐	A description of all covariates tested
☐	☒	A description of any assumptions or corrections, such as tests of normality and adjustment for multiple comparisons
☐	☒	A full description of the statistical parameters including central tendency (e.g. means) or other basic estimates (e.g. regression coefficient) AND variation (e.g. standard deviation) or associated estimates of uncertainty (e.g. confidence intervals)
☒	☐	For null hypothesis testing, the test statistic (e.g. *F*, *t*, *r*) with confidence intervals, effect sizes, degrees of freedom and *P* value noted<br>*Give P values as exact values whenever suitable.*
☒	☐	For Bayesian analysis, information on the choice of priors and Markov chain Monte Carlo settings
☒	☐	For hierarchical and complex designs, identification of the appropriate level for tests and full reporting of outcomes
☐	☒	Estimates of effect sizes (e.g. Cohen's *d*, Pearson's *r*), indicating how they were calculated

Our web collection on statistics for biologists contains articles on many of the points above.

Software and code

Policy information about availability of computer code

Data collection	No software was used to collect the data.
Data analysis	Data analysis was done in Python 2.7 and 3.9 with use of various packages. However, all algorithms and code for analysis were custom-designed and are available at https://github.com/tjparmer/influence_maximization_BNs

For manuscripts utilizing custom algorithms or software that are central to the research but not yet described in published literature, software must be made available to editors and reviewers. We strongly encourage code deposition in a community repository (e.g. GitHub). See the Nature Portfolio guidelines for submitting code & software for further information.

Data

Policy information about availability of data

All manuscripts must include a data availability statement. This statement should provide the following information, where applicable:

- Accession codes, unique identifiers, or web links for publicly available datasets
- A description of any restrictions on data availability
- For clinical datasets or third party data, please ensure that the statement adheres to our policy

Network data considered in this paper have been downloaded from the publicly accessible repositories https://github.com/rionbr/CANA/tree/master/cana/datasets and https://cellcollective.org

Human research participants

Policy information about studies involving human research participants and Sex and Gender in Research.

Reporting on sex and gender	n/a
Population characteristics	n/a
Recruitment	n/a
Ethics oversight	n/a

Note that full information on the approval of the study protocol must also be provided in the manuscript.

Field-specific reporting

Please select the one below that is the best fit for your research. If you are not sure, read the appropriate sections before making your selection.

☒ Life sciences ☐ Behavioural & social sciences ☐ Ecological, evolutionary & environmental sciences

For a reference copy of the document with all sections, see nature.com/documents/nr-reporting-summary-flat.pdf

Life sciences study design

All studies must disclose on these points even when the disclosure is negative.

Sample size	For biological networks, we considered all networks available in the Cell Collective (at the time this was 77). For Random Boolean Networks (RBNs), we used 10 different models for network sizes N=10, 20, 30,..., 100 to add variability while keeping computational time feasible. For simulation studies, we used R=100 simulations to explore the state space available while also keeping computational time feasible.
Data exclusions	Three cell collective networks were excluded due to missing SBML information, leaving 74 that we analyzed; these were the only exclusions. The 74 we did analyze are those found at https://github.com/rionbr/CANA/tree/master/cana/datasets/cell_collective as of Mar 12, 2021.
Replication	Many results are deterministic (e.g., mean-field estimates) and the analyses were run multiple times to ensure that the values never changed. Simulations were also run multiple times to ensure that mean values were similar across different selections.
Randomization	We did not have different experimental groups. Any randomization (for example, the starting conditions for simulations) was based off of the standard random library in python.
Blinding	This is not applicable because we didn't have any group allocation.

Reporting for specific materials, systems and methods

We require information from authors about some types of materials, experimental systems and methods used in many studies. Here, indicate whether each material, system or method listed is relevant to your study. If you are not sure if a list item applies to your research, read the appropriate section before selecting a response.

Materials & experimental systems

n/a	Involved in the study	
☒	☐	Antibodies
☒	☐	Eukaryotic cell lines
☒	☐	Palaeontology and archaeology
☒	☐	Animals and other organisms
☒	☐	Clinical data
☒	☐	Dual use research of concern

Methods

n/a	Involved in the study	
☒	☐	ChIP-seq
☒	☐	Flow cytometry
☒	☐	MRI-based neuroimaging
